# Evaluation of the effect of adding micro-hydroxyapatite and nano-hydroxyapatite on the microleakage of conventional and resin-modified Glass-ionomer Cl V restorations

**DOI:** 10.4317/jced.53216

**Published:** 2017-02-01

**Authors:** Farahnaz Sharafeddin, Negar Feizi

**Affiliations:** 1 Professor, Department of Operative Dentistry, Biomaterial Research Center, School of Dentistry, Shiraz University of Medical Sciences, Shiraz, Iran; 2 Postgraduate Student, Department of Operative Dentistry, School of Dentistry, Shiraz University of Medical Sciences, Shiraz, Iran

## Abstract

**Background:**

Pulpal reaction to restorative materials depends on marginal microleakage, which is a dynamic phenomenon that allows bacteria and fluids to traverse across the tooth-restoration interface. Glass-ionomer cement (GIC) exhibits low microleakage due to direct bonding to tooth structures. Hydroxyapatite (HAP) based on the similarity with tooth structure may decrease the microleakage. The aim of this *in vitro* study was to evaluate marginal microleakage of a mixture of conventional and resin-modified glass-ionomer (RMGI) with micro- and nano-HAP.

**Material and Methods:**

In this *in vitro* study, 30 non-carious extracted human third molar teeth were used. Standard Cl V cavities were prepared on the buccal and lingual surfaces. The cavities were restored in six experimental groups as follows: group 1, conventional glass-ionomer cement (CGIC); group 2, CGIC with micro-HAP; group 3, CGIC with nano-HAP; group 4, RMGI; group 5, RMGI with micro-HAP; group 6, RMGI with nano-HAP. The restorations were finished and polished. The teeth were coated with nail polish, sealed with sticky wax, thermocycled and placed in a solution of 2% basic fuchsine for 24 hours. The teeth were sectioned and microleakage was measured. Kruskal-Wallis, Man-Whitney and Wilcoxon tests were used for data analysis (*P*<0.05).

**Results:**

The data analysis revealed significantly lower microleakage in groups 5 and 6 at both occlusal and gingival margins. Also in these two groups the gingival microleakage was significantly lower than occlusal margin (*P*=0.009 and *P*=0.001 respectively), but in groups 1(CGIC) and 3(CGIC+ nano-HAP) and 4(RMGI) the microleakage of occlusal margin were significantly lower than that of gingival margin (*P*=0.001, *P*=0.007 and *P*=0.001 respectively).

**Conclusions:**

Mixing RMGI with nano-HAP and micro-HAP resulted in lower microleakage.

** Key words:**Glass-ionomer, micro-hydroxyapatite, microleakage, nano-hydroxyapatite.

## Introduction

Erosions, carious lesions or abfractions in cervical areas can be restored with several restorative materials (1). Restoring cervical lesions has always been a challenge as the margins of the cavity may be placed in dentin, enamel or cementum (2). Most probably the gingival margin of Cl V cavities ends up in dentin or cementum. Bonding of composite resin to both of these substrates is not as reliable as bonding to enamel (3). Polymerization shrinkage of composite resin is another disadvantage that gives rise to marginal gaps (4). In addition, in some cases this part of the cavity is not accessible; therefore, it cannot be isolated well. As a result, administration of a restorative material with good stability in an aqueous environment, such as conventional glass ionomer cement (CGIC) and resin modified glass ionomer RMGI (5), can decrease marginal gaps (6).

Glass ionomer cement (GIC) is one of the best materials for this purpose due to its good properties like direct bonding to tooth structure, biocompatibility and anti-cariogenic activity as a result of fluoride release (7-9). Lower shrinkage during setting of GIC leads to lower degrees of marginal microleakage in comparison with composite resin (10). Therefore, GICs can be promising materials for restoration of the whole cavity or for use in the sandwich technique.

Many studies have demonstrated that the pulpal reaction to restorative materials is dependent on marginal microleakage, which is a dynamic phenomenon allowing bacteria, fluids, molecules and ions to cross the tooth restoration interface (2).

In the cervical region of teeth we should use a restorative material which can withstand the shear and flexural forces during loading of the tooth. As flexural and tensile strengths of GIC is in a range that can tolerate these loads (11) and due to its fluoride release and water absorption that result in lower gap formation, (10) it can be the material of choice for cervical lesions and root caries.

In order to overcome some shortcomings of GIC different materials have been incorporated into glass powder, including silver, gold and stainless steel. These reinforced GICs exhibit reduced abrasion but their poor aesthetic limits their use (7). Zirconia and its oxides have been used to improve the strength of glass-ionomer due to their dimensional stability and toughness (6). In addition, materials like bioactive glass (BAG) and hydroxyapatite (HAP) have been added to GIC (12,13). It was reported that some mechanical properties like diametral and flexural strength, fracture resistance, bond strength and compressive strength improved by mixing the glass powder with bioceramics (14,15). They can also enhance the flexural strength of demineralized dentin by remineralization (16,17).

RMGI is one result of attempts for improving the problems like low early mechanical strength of CGIC (18).

The aim of this study was to evaluate marginal microleakage of CGIC and RMGI when mixed with micro- and nano-HAP.

## Material and Methods

Following approval of the research protocol by the university ethic committee (by the code of CT-P-9378-7158), thirty extracted human maxillary and mandibular molar teeth were used for the purpose of this experimental study. All the debris was removed by a scaling instrument and a rubber cup with slurry of pumice. The teeth were disinfected with 0.2% thymol solution for 48 hours (19) and stored in distilled water. Teeth without any caries, cracks, cervical erosion or attrition were selected. We used new straight fissure burs (Diamond Fissure 330, SS White, Washington, USA) for every five preparations in a high-speed handpiece with air/water spray to prepare standard class V cavities (5 mm in length, 3 mm in width and 2 mm in depth) (2) on the buccal and lingual surfaces of each tooth. A periodontal probe was used to confirm the dimensions of the cavities. The occlusal margin of all the cavities was placed in enamel but the gingival margins were placed 1 mm below CEJ in the cementum. The teeth were randomly divided into 6 equal groups of 10 cavities each.

The brand names, manufacturers and composition of all the materials are presented in  Table 1.

**Table 1:**
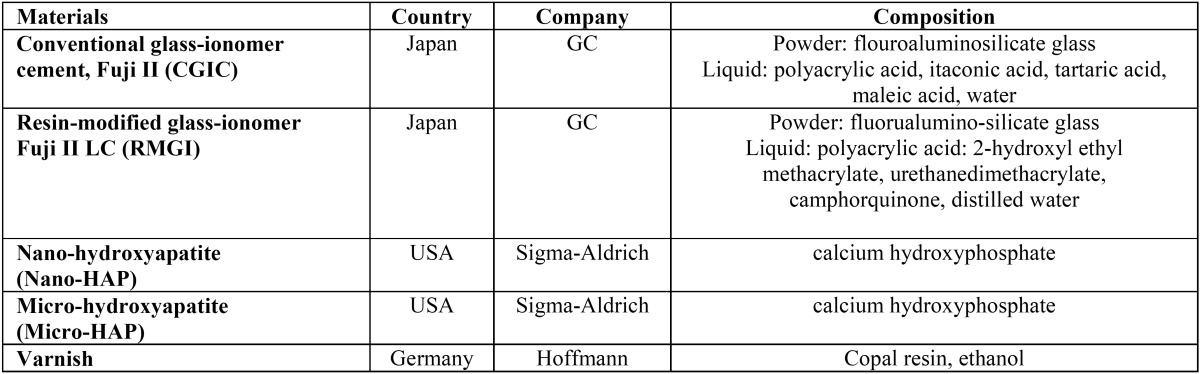
The materials used in the study.

To prepare the mixtures of filling materials, CGIC (GC, Tokyo, Japan), RMGI (GC, Tokyo, Japan), micro-HAP (Sigma-Aldrich, St. Louis, USA) and nano-HAP (Sigma-Aldrich, St. Louis, USA) were weighed carefully using a weighing machine accurate to 0.0001 g (A&D, GR+360, Tokyo, Japan). Then a mixture of CGI and micro-HAP (25% by weight) (20) was prepared. Another mixture was prepared by adding 25% by weight of nano-HAP to CGIC. The same process was repeated for RMGI. To have a uniform mixture, an amalgamator (Ultramat, SDI, Bayswater, Victoria, Australia) was used. These four mixtures were mixed in the amalgamator using clean amalgam capsules for 20 seconds separately (18).

Before restoring the cavities, all the cavities were rinsed with water and air-dried but not desiccated. The cavities were restored in the following groups:

-Group 1, the control group: CGIC powder was mixed with the liquid with the ratio recommended by the manufacturer (2.7). It was mixed on a clean cold slab with a plastic spatula within 30 seconds. The cavity was filled with CGIC using a plastic instrument. A transparent Mylar matrix (Fintrec transparent matrix, M-TP, Pulpdent Corporation, Watertown, MA, USA) was adapted over the restoration. After 5 minutes and 30 seconds from the start of mixing when the material was set, a thin layer of copal varnish (Hoffmann, Berlin, Germany) was applied on its surface and gently air-dried for 30 seconds.

-Group 2, CGIC+ micro-HAP: The prepared powder was mixed with the liquid of CGIC with a ratio of 2.7. Restorative steps were just like group one.

-Group 3, CGIC+ nano-HAP: The mixing and restorative procedures were carried out as described in the previous groups. The powder-to-liquid ratio was 2.7.

-Group 4, RMGI: Ten cavities of this group were restored with RMGI. The powder and liquid were mixed with a ratio of 3.2, according to the manufacturer’s instructions in 25 seconds. After filling the cavity, a mylar matrix was placed on its surface and cured with an LED light-curing unit (BlueLex, GT1200, Monitex, San-Chong, Taiwan) with a light intensity of 1200 mW/cm for 20 seconds. Then a layer of varnish was applied and dried.

-Group 5, RMGI+ micro-HAP: The restorative material in this group was the mixture of RMGI and micro-HAP. The powder-to-liquid ratio was 3.2. All the procedures were exactly the same as those in group 4.

-Group 6, RMGI+ nano-HAP: The cavities of the last group were restored with a mixture of RMGI and nano-HAP, which was mixed with RMGI liquid with a powder-to-liquid ratio of 3.2. The material was cured for 20 seconds with an LED light-curing unit.

All the teeth were stored in deionized water in separate tanks for 24 hours at room temperature. Then the surface of all the restorations were finished with finishing burs and polished carefully with polishing disks (Polipro Disks, Premier Dental Products, Plymouthmeeting, Pennsylvania, USA) from course to fine. To simulate oral condition, a thermocycling machine (TC-300, Vafaei Industrial, Tehran, Iran) was use for 1000 cycles (2) at 5±2/55±2 ºC with a dwell time of 30 seconds (10). The surface of all the teeth was covered with two layers of nail varnish except for 1 mm from the margins of the restoration. During application of nail polish, a moist cotton pellet was placed on it in order to protect the restoration from desiccation. The apices were sealed with sticky wax. Then they were stored in 2% basic fuchsine solution (Merck, Germany) for 24 hours at room temperature. After removal of the specimens from the dye solution the superficial dye was washed with tap water and cleaned with rubber cup and pumice slurry. All the teeth were sectioned longitudinally in a buccolingual direction in the middle of the restoration using a diamond disk (Diamond disk, Microdont, Brazil) in a nonstop cutting machine (Demco E96, CMP Industries, NY, USA) under a water spray.

The sectioned teeth were examined under a stereomicroscope (Estscope BS-3060, Best Scope, Beijing, China) at ×25. Two blinded examiners measured the extent of dye penetration at both gingival and occlusal margins using these microleakage scores: 0 = no dye penetration; 1 = dye penetration between the restoration and the tooth up to one-third of the distance between the tooth surface and the axial wall; 2 = dye penetration extending beyond one-third of the distance up to two-thirds of the distance between the tooth surface and the axial wall; 3 = dye penetration extending up to two-thirds of the distance between the tooth surface and the axial wall; 4 = dye penetration reaching the axial wall; and 5 = dye penetration reaching the entire axial wall (21).

Data were described using mean ranks and medians×.

Kruskal-Wallis H and Mann-Whitney U tests were used to compare the microleakage values between the groups. We also employed Wilcoxon signed-rank test to compare microleakage values between occlusal and gingival margins. SPSS 18.U (Chicago, IL., USA) was employed for data analysis; *P*<0.05 was considered statistically significant.

## Results

The mean ranks and medians are illustrated in  Table 2. According to the results, occlusal and gingival microleakage in group 6 (RMGI+ nano-HAP) was lower in comparison with other 5 groups.

**Table 2:**
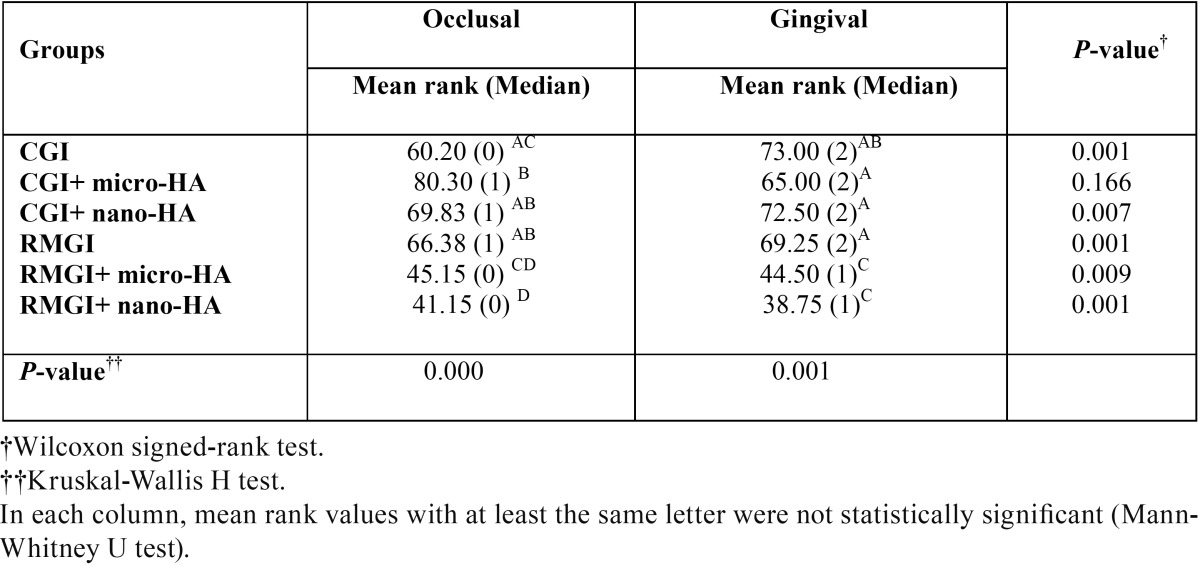
Comparison of microleakage in all the groups [Mean rank (Median)].

Also occlusal microleakage of group 6 was significantly lower than that in group 1 (CGIC), group 2 (CGIC+ micro-HAP), group 3 (CGIC+ nano-HAP) and group 4 (RMGI) (*P*<0.001).

Group 5 exhibited significantly lower microleakage at occlusal margins in comparison with groups 2, 3 and 4.

Gingival microleakage in groups 5 and 6 was significantly lower than that in groups 1, 2, 3 and 4 (*P*=0.001). Occlusal and gingival microleakage in group 6 was lower than group of 5 but it was not statistically significant.

The most severe microleakage was the occlusal microleakage in group 3 (CGIC+ nano-HAP) and the lowest percentage was the occlusal microleakage in group 6 (RMGI+ nano-HAP).

Wilcoxon signed-rank test showed that occlusal and gingival microleakage in all the groups, except for group 2, was significantly different. Microleakage of gingival margin was higher than that of the occlusal margin in groups 1(*P*=0.001), 3(*P*=0.007) and 4(*P*=0.001). In the other groups gingival microleakage was lower than occlusal microleakage.

**Figure 1:**
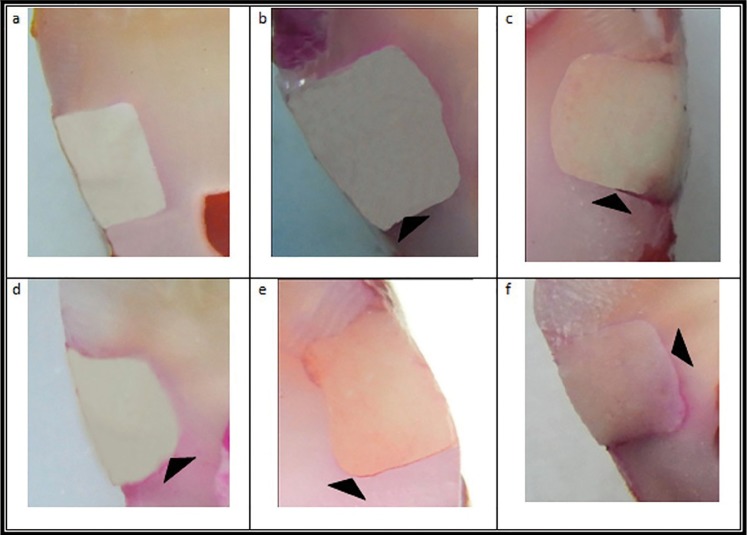
a: 0 = no dye penetration; b: 1 = dye penetration between the restoration and the tooth up to one-third of the distance between the tooth surface and the axial wall; c: 2 = dye penetration extending beyond one-third of the distance up to two-thirds of the distance between the tooth surface and the axial wall; d: 3 = dye penetration extending up to two-thirds of the distance between the tooth surface and the axial wall; e: 4 = dye penetration reaching the axial wall; and f; 5 = dye penetration reaching the entire axial wall.

Figure 1 illustrates the sectioned teeth which were evaluated at ×25.

**Figure 2:**
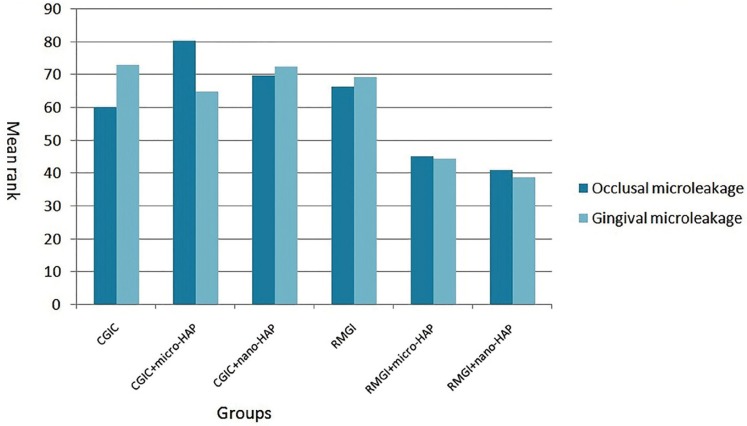
Occlusal and gingival microleakage in experimental groups (Mean rank).

Figure 2 showes the mean rank of the microleakage of experimental groups.

## Discussion

The quality of marginal seal obtained by restorative materials has always been a concern in dentistry. The marginal seal is evaluated by microleakage test. Several techniques are used to evaluate microleakage like Rhodamine B, (4) methylene blue, basic fuchsine and silver nitrate (22) are used. Silver nitrate particles are smaller than bacterial sizes so the leakage with the use of silver nitrate *in vitro* may be more than that *in vivo* (5). Methylene blue dye has some disadvantages like dissolution during clearing process (23). The most popular dye for the test is basic fuchsine (0.5-2%). It is easy to manipulate, is economical and does not require any complex laboratory equipment (24). Therefore, in this study 2% basic fuchsine solution was used.

One cause of microleakage is the difference between coefficients of thermal expansion of dentin and the restorative material (5). Thermal stress in the oral environment can cause periods of expansion and contraction in the restorative material and dentin. When coefficients of thermal expansion are different the stresses lead to gap formation. These coefficients are similar for GIC and dentin. Thermocycling is the only method for simulation of thermal stresses in the oral environment. In this study 1000 thermocycles were used to simulate long-term clinical use of the restoration.

The polyacrylic acid in the liquid of GIC can decalcify the dentin surface so Moshaverinia *et al.* suggest no pretreatment on tooth structure before restoring with GIC (15). In the present study no surface treatment was implemented prior to the application of GIC.

Mature enamel and dentin contain 90-92% and 50% of HAP by volume, respectively. When GIC is applied on enamel and dentin, a chemical reaction occurs between carboxylic groups of polyacrylic acid and the calcium ions in HAP structure. Adding HAP with its excellent biological activity and crystal structure similar to those of dental apatite to GIC can increase the mechanical properties and bond strength to dentin (13). Additionally, RMGI contains polymerizing resin that improves the polymerization process. Enan *et al.* (25) reported that adding nano-HAP to RMGI has a positive effect on microleakage around orthodontic bands. HAP particles, due to their role in the interaction between the powder and liquid of GIC, may release ions which participate in the acid-base reaction. Presence of ions in the tooth structure-restorative material interface may lead to the formation of more hydrogen and ionic bonds (13). It seems that the better bonding is the cause of lower microleakage. Moshaverinia *et al.* (13) reported elevated bond strength when HAP and flouro-HAP were added to GIC. Lucas *et al.* (15) showed higher bond strength at 7- and 56-day intervals and long-term maintenance of bond strength after adding HAP to GIC. According to the results, occlusal and gingival microleakage in groups 5 and 6 was significantly lower than that in other groups, which can be explained by the higher bond strength of the mixture to tooth structure, and is the result of HAP participation in chemical reactions.

Moreover in a chemical reaction the particle size is important and by decreasing the size of the particles greater surface is available for interaction. Although it was predicted that groups with nano-HAP would exhibit lower microleakage in comparison with groups containing micro-HAP, no significant differences were seen between groups 5 and 6 and between groups 2 and 3. According to the result, it seems that in the case of HAP the particle size cannot affect the reaction significantly.

A great concern in bonded restorations is microleakage in the restoration dentin interface. Xie *et al.* (26) reported that the micro-leakage of flowable composite, compomer and GIC was not significantly different at occlusal and gingival margins. Also Mazaheri *et al.* (27) did not show significant differences in microleakage of glass-ionomer cement at enamel and dentin margins in primary teeth. In contrast, the results of the present study showed that microleakage values of occlusal margins in all the experimental groups were lower than those at gingival margins. It is in accordance with the common knowledge that the adhesion to enamel is more effective than that to dentin (28) and can be related to the higher mineral content of enamel.

One study suggest 45-degree beveling on enamel for resin-modified restorative material to decrease microleakage (29). It seems that by beveling the margin a greater surface becomes available to interact with the material. This factor along with stronger bonding, based on the higher content of minerals in the enamel, can results in lower microleakage. In this study no bevels were used. It is possible that by beveling the enamel in groups 5 and 6 no microleakage might have been detected, but in the present study such correlation is not certain.

In a study only 200 thermocycles (29) were used but in this study 1000 thermocycles were implemented. Due to lower bond strength of GIC to dentin and cementum, it might be affected by thermocycling more than that of enamel. It can be another reason for higher dye penetration at gingival margin.

Previous studies showed that RMGI exhibits lower microleakage in comparison with CGIC (22). In our study RMGI did not exhibit statistically lower microleakage in comparison with CGIC. Not using any retreatments seems to be the reason for this similarity. In addition, it is possible that by applying the conditioner the microleakage in all the groups decreased to some extent due to formation of greater micromechanical bonds (5). So it is highly recommended to evaluate the effect of different pretreatments on marginal microleakage of GIC restorations.

The results of the current study showed that although the occlusal microleakage of CGIC did not change significantly by adding micro- and nano-HAP, the gingival microleakage increased. It seems that in the case of CGIC, although the mechanical properties improved by adding HAP, no positive changes could be seen in marginal microleakage.

It is highly recommended that the effect of adding different amounts of HAP to RMGI be evaluated and clinical research be designed to evaluate this mixture or at least simulate oral conditions by using cyclic loading and acidic liquids along with thermocycling.

## Conclusions

Within the limitations of this study it was concluded that adding HAP in both micro- and nano-sizes to RMGI decreased marginal microleakage at enamel and dentin/cementum interface. Both sizes of HAP did not change the microleakage in combination with CGIC.
